# The role of ACE2, angiotensin-(1–7) and Mas1 receptor axis in glucocorticoid-induced intrauterine growth restriction

**DOI:** 10.1186/s12958-017-0316-8

**Published:** 2017-12-29

**Authors:** Elham Ghadhanfar, Aseel Alsalem, Shaimaa Al-Kandari, Jumana Naser, Fawzi Babiker, Maie Al-Bader

**Affiliations:** 1 Department of Physiology, Faculty of Medicine, Kuwait University, Kuwait City, Kuwait; 2 Faculty of Medicine, Kuwait University, Kuwait City, Kuwait

**Keywords:** Dexamethasone, IUGR, Ang-(1–7), ACE2, MME, Mas1 receptor

## Abstract

**Background:**

Plasma and urine levels of the potent vasodilator Ang-(1–7) are elevated in mid and late pregnancy and are correlated with elevated placental angiogenesis, fetal blood flow, and rapid fetal growth. We hypothesized that Ang-(1–7), its receptor (Mas1) and the enzymes involved in Ang-(1–7) production (ACE2 and Membrane metallo-endopeptidase; MME) are down regulated in response to glucocorticoid administration contributing to IUGR.

**Methods:**

Pregnant female Sprague–Dawley rats were injected with dexamethasone (DEX; 0.4 mg/kg/day) starting from 14 day gestation (dg) till sacrifice at 19 or 21 dg while control groups were injected with saline (*n* = 6/group). The gene and protein expression of ACE2, MME, Ang-(1–7) and Mas1 receptor in the placental labyrinth (LZ) and basal zones (BZ) were studied.

**Results:**

DEX administration caused a reduction in LZ weight at 19 and 21 dg (*p* < 0.001). IUGR, as shown by decreased fetal weights, was evident in DEX treated rats at 21 dg (*p* < 0.01). ACE2 gene expression was elevated in the LZ of control placentas at 21 dg (*p* < 0.01) compared to 19 dg and DEX prevented this rise at both gene (*p* < 0.01) and protein levels (*p* < 0.05). In addition, Ang-(1–7) protein expression in LZ was significantly reduced in DEX treated rats at 21 dg (*p* < 0.05). On the other hand, Mas1 and MME were upregulated in LZ at 21 dg in both groups (*p* < 0.05 and *p* < 0.001, respectively).

**Conclusion:**

The results of this study indicate that a reduced expression of ACE2 and Ang-(1–7) in the placenta by DEX treatment may be responsible for IUGR and consequent disease programming later in life.

## Background

Administration of glucocorticoids (Betamethasone and/or Dexamethasone) to pregnant women endangered by premature labor is a standard clinical procedure beneficial in terms of maturation of fetal lungs and prevention of serious fetal respiratory problems [1–4]. Despite the beneficial effects of glucocorticoids on fetal lungs, concomitant side effects were observed. Dexamethasone is a poor 11β-hydroxysteroid dehydrogenase 2 substrate and its administration in rats between gestational days 13 and 20 induces intrauterine growth restriction (IUGR) and decreases placental mass by approximately 50% [5–7]. Reduced fetal growth with maternal administration of synthetic glucocorticoids has been observed in several species including humans [8–10], and this was associated with reduced placental growth [8, 10].

In rats, the placenta consists of two discrete zones that differ in both function and morphology; the basal and labyrinth zones. The basal zone is the site of placental hormone production during mid to late pregnancy [11] with both trophoblast and maternal vessels, but no fetal vessels [12]. The labyrinth zone is close to the fetal side and includes trophoblast cells as well as maternal and fetal vessels. It is the main area involved in maternal-fetal hemotrophic exchange of nutrients and waste-products [12, 13]. In late pregnancy, the basal zone undergoes apoptosis whereas the labyrinth zone experiences rapid and intense angiogenesis and grows substantially [14, 15]. These changes in the labyrinth zone promote expansion of maternal blood space and enhance fetal capillary development which in turn could be the reason behind the remarkable increase in placental efficiency in late pregnancy [13, 16–18]. Factors modifying labyrinth zone vascularization and development in late pregnancy may compromise fetal growth and largely contribute to IUGR.

**Fig. 1:**
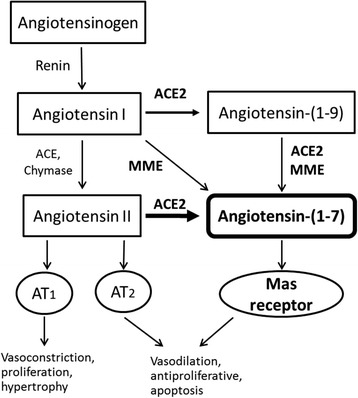
Components of the renin-angiotensin system (RAS); Ang II and Ang-(1–7), their receptors and the enzymes involved in the production pathways. Bold arrow shows the major pathway. ACE, Angiotensin 1-converting enzyme; ACE2, Angiotensin 1-converting enzyme2; MME, Membrane metallo-endopeptidase; AT1, Angiotensin II type 1 receptor; AT2, Angiotensin II type 2 receptor. Modified from [41]

The renin angiotensin system (RAS) has various homeostatic functions in the body including control of body sodium and water content and vascular smooth muscle tone. The functions of RAS are achieved through the balance between two main functional peptides the vasoconstrictor Angiotensin II (Ang II) and the vasodilator Angiotensin 1–7 (Ang-(1–7)) as well as the abundance of their receptors; Ang II type 1 and type 2 receptors (AT1 and AT2) and Ang-(1–7) Mas1 receptor (Fig. 1). The precursor for RAS peptides (angiotensinogen) is expressed in the liver. In response to a decrease in blood pressure, renin converts angiotensinogen to angiotensin I. Angiotensin I is a major substrate for angiotensin converting enzyme (ACE) and chymases producing the potent vasoconstrictor Ang II. Despite the well-known physiological role of Ang II in maintaining blood pressure, dysregulation of Ang II production contributes to the elevation of blood pressure in essential hypertension and preeclampsia. The other component of RAS, Ang-(1–7), which has counter regulatory functions to Ang II is a heptapeptide that can be produced by three possible pathways. The major pathway is by cleavage of Ang II via Angiotensin I-converting enzyme 2 (ACE2) [19]. Another pathway involves the formation of Ang-(1–7) directly from Ang I by Membrane Metallo-Endopeptidase (MME) [20], also known as Neprilysin, neutral endopeptidase (NEP) or cluster of differentiation (CD10). In the third pathway, angiotensin-1 is converted by ACE2 into Ang-(1–9) which is subsequently converted into Ang-(1–7) by Angiotensin I-converting enzyme (ACE) and/or NEP [21, 22]. Ang-(1–7) is a vasodilator and an endogenous ligand for Mas1 receptors. Activation of Mas1 receptors leads to nitric oxide release and subsequent hyperpolarization via activation of potassium channels [23].

Reports indicate that the activity of RAS is progressively enhanced during normal pregnancy suggesting that this system is essential for the maintenance and development of healthy pregnancies. In early pregnancy, circulating levels of angiotensinogen, renin activity and Ang II are elevated [24, 25]. Binding of Ang II to its receptor AT1 stimulates decidualization and rapid trophoblast proliferation in early pregnancy indicating a role of Ang II in implantation [26–28]. On the other hand, in mid to late pregnancy, an increase in urinary and plasma levels of Ang-(1–7) and ACE2 were found as well as an increase in local placental/uterine production and activity of ACE2 suggesting a role for Ang-(1–7) in the enhancement of placental-fetal blood flow and rapid fetal growth in mid to late gestation [29–33].

The effect of prenatal glucocorticoid treatment on the levels of ACE2, MME, Ang-(1–7) and its receptor Mas1 has not been studied yet in rat placenta and the association with IUGR, if any, is to be identified. We hypothesize that ACE2, MME, Ang-(1–7) and its receptor (Mas1) may be down regulated in response to glucocorticoid administration contributing to reduced blood flow and supply of nutrients to the fetus ultimately resulting in IUGR development and programming of diseases later in life.

## Methods

### Animal model and sample preparation

All procedures used in this study were approved by the Animal Welfare Committee at Kuwait University Health Sciences Center. Male and female Sprague Dawley rats were mated overnight. A vaginal smear was taken the following day and presence of sperm confirmed pregnancy (day 0). Pregnant rats were then housed individually in Plexiglas cages undisturbed until dexamethasone injection regimen. Half the number of pregnant rats (*n* = 12) received a daily intraperitoneal (i.p.) injection of dexamethasone (0.4 mg/kg) dissolved in saline from 14 days gestation (dg) until 21 dg. The control group of pregnant rats (*n* = 12) received daily i.p. injections of the vehicle (saline) on the same gestational days.

Pregnant dams were sacrificed by cervical dislocation on 19 (*n* = 6) and 21 dg (n = 6). Fetuses and placentae were detached and weighed. The placenta was further dissected into the basal and labyrinth zones and samples were snap frozen in liquid nitrogen and subsequently stored at −70 °C. Detection of ACE2, Mas1 and MME mRNAs and ACE2, Ang-(1–7), Mas1 and MME proteins was performed using real time PCR (Ret-PCR) and Western blotting followed by immunodetection, respectively.

### Gene studies using ReT-PCR

Total RNA was extracted from both the basal and labyrinth zones using Trizol (Invitrogen) and stored at −70 °C [17]. RNA concentration and purity was assessed by measuring the absorbance using Epoch Microplate Spectrophotometer (BioTek) at 260 and 280 nm wave lengths. If the 260/280 absorbance ratio was less than 1.7, the sample was excluded. Samples passing the purity test were run on 1% agarose gel to confirm RNA integrity which is indicated by the 28S band having double the size of 18S rRNAs when seen under UV after staining with ethidium bromide.

All samples were DNase treated before reverse transcription as previously described [17]. DNase-treated samples were then mixed with random hexamer primers and heated at 70 °C for 10 min then chilled on ice. Samples were divided equally into 2 halves and the following mixture was added to all tubes: 1× first strand buffer, 5 mM DTT and 500 μM dNTP mix. The enzyme Superscript II RNase H- reverse transcriptase was added to one half (RT+ reaction), while water replaced the enzyme in the other half of the sample (RT- reaction; control for the existence/absence of genomic DNA). All tubes were incubated at room temperature for 10 min then in the thermomixer at 42 °C for 50 min. The reaction was terminated by heating at 70 °C for 15 min after which samples were stored at −20 °C until needed for ReT-PCR.

The ReT-PCR reaction was run in duplicates in MicroAmp optical 96-well reaction plates (#4306737, Applied Biosystems). The reaction mix included the following: 12.5 μl of 2× Taqman universal master mix (#4369016, Applied biosystems), 1.25 μl target gene primer set (ACE2, Mas1, MME), 1.25 μl reference gene primer set (B-actin) in addition to 1 μl of the sample cDNA and the final volume was brought to 25 μl with distilled deionized water. The plate was sealed and cycled in a 7500 ReT-PCR system (Applied Biosystems) as follows: 1 cycle of 2 min at 50 °C; 1 cycle of 10 min at 95 °C, followed by 60 cycles each consists of 15 s at 95 °C to denature the DNA strands and 1 min at 60 °C to allow primer annealing and extension. The gene expression of Ang-(1–7) was not studied because no specific primers exist for Ang-(1–7) as all angiotensin peptides are formed from one common inactive precursor peptide which is angiotensinogen. Studying the gene expression of angiotensinogen does not give a direct conclusion about Ang-(1–7) expression.

### Protein studies using western blotting followed by Immunodetection

Protein expression of ACE2, Ang-(1–7), Mas1 and MME was studied using Western blotting followed by immunodetection. Placental basal and labyrinth zones were homogenized in ice-cold homogenization buffer containing glycerol and protease inhibitors. Protein concentration was estimated using Versa max microplate reader at a wave length of 562 nm. Loading buffer was added to samples (60 μg protein loaded) and rainbow marker (14,300–220,000 Da, Amersham Pharmacia Biotech Ltd., U.K.). Tubes were boiled for 5 min, then cooled on ice and centrifuged for 1 min. Electrophoresis was performed using SDS-PAGE gel (SDS PAGE; 5–14% polyacrylamide gradient gels). Protein was transferred from the gels to PVDF membranes at a stable current of 300 mA overnight at 4 °C. The efficiency of transfer was confirmed by staining the gel with Coomassie blue stain. After transfer was confirmed, membranes were blocked for 1 h at room temperature with 10% non-fat dry milk in TBS-T. They were rinsed twice with TBS-T then washed 2 times for 10 min. The primary antibody (Table 1) diluted in non-fat dry milk in TBS-T was then added to the membranes which were incubated overnight at 4 °C. Next day, membranes were washed and incubated with the secondary antibody (Table 1) for 1 h and 30 min at room temperature then rinsed and washed several times as described before [17]. Protein bands were detected by chemiluminescence using ECL-Plus kit (Amersham Pharmacia Biotech Ltd.). The membranes were re-probed with the anti-actin antibody as an endogenous control after stripping. Briefly, membranes were incubated in the stripping buffer (100 mM 2-Mercaptoethanol, 2% SDS, 62.5 mM Tris–HCl, pH 6.7) at 50 °C for 30 min with intermittent shaking. The membranes were then washed twice for 10 min in TBS-T after which membranes were incubated with ECL Plus and exposed to film which showed no bands indicating the removal of primary and secondary antibodies. Then membranes were blocked with 10% non-fat dry milk in TBS-T for 1 h and re-probed with anti-actin antibody. The specificity of the primary antibodies was confirmed by eliminating the primary antibodies and probing the membranes with the secondary antibodies only.

**Table 1:** Primary and secondary antibodies used for Western blotting

Primary Antibody	Dilution	Catalogue Number	Company	Secondary Antibody	Dilution	Catalogue Number	Company
Ang-(1–7)	1:200	PAS085Ra01	Cloud-Clone Corporation	Anti-rabbit from donkey	1:10,000	NA934	Amersham
MME	1:250	Ab126593	Abcam	Anti-rabbit from donkey	1:10,000	NA934	Amersham
ACE2	1:250	Sc-20,998	Santa Cruz	Anti-rabbit from goat	1:5000	Sc-2004	Santa Cruz
Mas1	1:100	Sc-390,453	Santa Cruz	Anti-mouse from sheep	1:20,000	NA931	Amersham
B-actin	1:5000–1:20,000	MAB1501R	Millipore corporation	Anti-mouse from sheep	1:20,000	NA931	Amersham

Ang-(1–7), angiotensin (1–7); MME, membrane metallo-endopeptidase; ACE2, Angiotensin 1-converting enzyme2; Mas1, mas-related G-protein coupled receptor A

The distance travelled by the marker proteins and bromophenol blue was measured and a standard curve was plotted to extrapolate the target and endogenous control band sizes which were determined from the equation of the line plot.

### Statistical analysis

All data are shown as mean ± SEM. Data were tested for statistical significance by two way analysis of variance (ANOVA) followed by least significant difference (LSD) post hoc analysis using SPSS software as described earlier [18]. A “p” value <0.05 was considered as the lowest level of significance.

## Results

### Effect of dexamethasone on litter number, body and organ weight, and placental efficiency

**Fig. 2:**
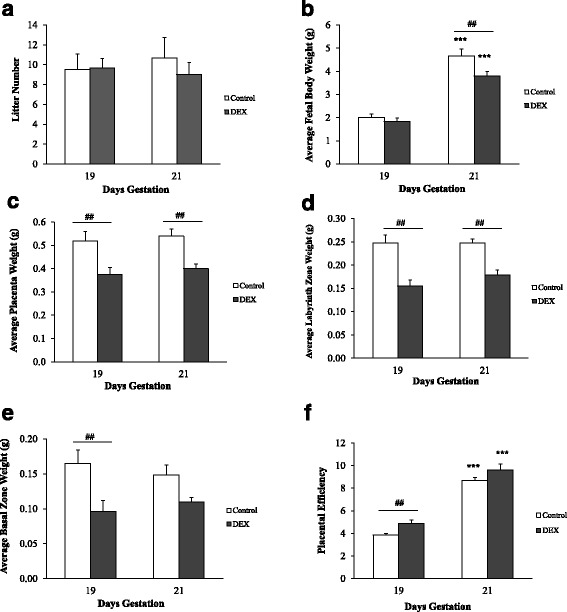
Gross data analysis of controls (open bars) and DEX-treated (solid bars) rats at 19 dg and 21 dg. Data analyzed were Litter number (**a**), average fetal body weight (**b**), placental weight (**c**), labyrinth (**d**) and basal (**e**) zones weights and placental efficiency (**f**). Error bars represent the mean ± SEM (*n* = 6 per group). ***p < 0.001 compared with 19 dg of same group. ##*p* < 0.01 compared with untreated rats at same gestational day

The litter number did not significantly vary between the groups (Fig. 2a). The average fetal body weight increased significantly with gestation in both control and DEX groups (*p* < 0.001), however, weights were significantly lower (*p* < 0.001) at 21 dg in the DEX group compared to control (Fig. 2b). There was a significant reduction in placental (*p* < 0.01) and labyrinth (*p* < 0.01) weights in the DEX group compared to the control group at both 19 dg and 21 dg (Fig. 2c & d). The basal weight was significantly less in the DEX group compared to the control group at 19 dg only (*p* < 0.01; Fig. 2e). The placental efficiency (calculated as the ratio between the weight of a fetus and the weight of a placenta) increased significantly with gestation (*p* < 0.001) and only showed significant difference between the control and experimental group at 19 dg (DEX > control; *p* < 0.01; Fig. 2f).

### Effect of dexamethasone on the gene expression of ACE2, Mas1, and MME in basal and labyrinth zones

**Fig. 3:**
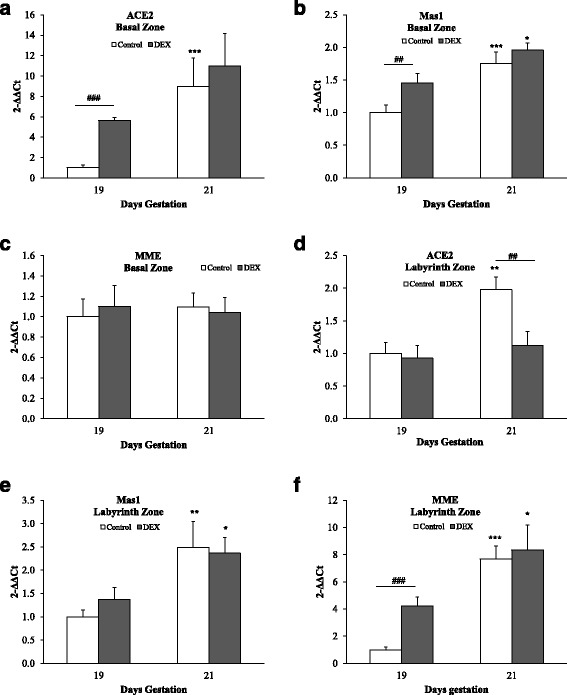
Quantitative real-time PCR analysis (2^-∆∆Ct^ values) of ACE2, Mas1 and MME gene expression in basal (**a**-**c**) and labyrinth (**d**-**f**) zones. Open bars denote controls while solid bars are DEX-treated rats. Error bars represent the mean ± SEM (*n* = 6 per group). *p < 0.05, **p < 0.01, ***p < 0.001 compared with 19 dg of same group. ##*p* < 0.01, ###*p* < 0.001 compared with untreated rats at the same gestational day

In the basal zone, the mRNA levels of ACE2 and Mas1 were significantly higher within the control groups at 21 dg compared to 19 dg (*p* < 0.001; Fig. 3a & b). The mRNA levels of Mas1 were also significantly higher in the DEX groups at 21 dg compared to 19 dg (*p* < 0.05; Fig. 3b). In addition, the mRNA levels of ACE2 and Mas1 were significantly higher in the DEX group at 19 dg compared to the controls (*p* < 0.001 and *p* < 0.01, respectively; Fig. 3a & b). No significant differences in MME expression were seen between groups nor with gestation (Fig. 3c).

In the labyrinth zone, the mRNA levels of ACE2, Mas1, and MME were significantly higher in the control groups at 21 dg compared to 19 dg (*p* < 0.01, *p* < 0.01, *p* < 0.001, respectively; Figs. 3D,E&F). The mRNA levels of Mas1 and MME were also significantly higher in the DEX groups at 21 dg compared to 19 dg (*p* < 0.05; Fig. 3e & f). In addition, the mRNA levels of ACE2 were significantly lower in the DEX group compared to the control group at 21 dg (*p* < 0.01; Fig. 3d). However, at 19 dg the mRNA levels of MME were significantly higher in the DEX group compared to the control group (*p* < 0.001; Fig. 3f).

### Effect of dexamethasone on the protein expression of ACE2, Ang-(1–7), MME, and Mas1 in labyrinth and basal zones

**Fig. 4:**
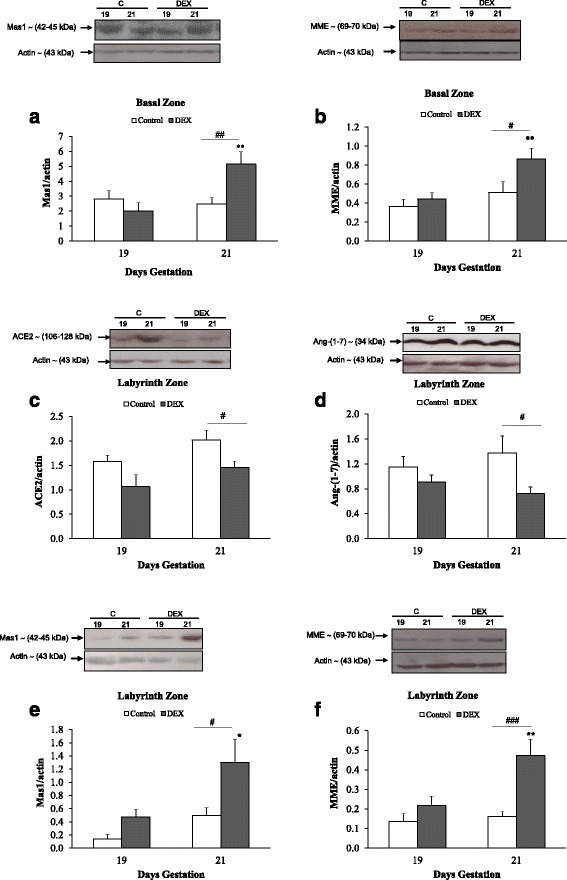
Western blot analysis of Mas1 receptor (**a**) and MME (**b**) in basal zone of the placenta. (**c**-**f**) represent the western blot analysis of ACE2, Ang-(1–7), Mas1 and MME in the placental labyrinth zone. Open bars denote controls while solid bars are DEX-treated rats. Error bars represent the mean ± SEM (*n* = 6 per group). *p < 0.05, **p < 0.01 compared with 19 dg of same group. #*p* < 0.05, ##*p* < 0.01, ###*p* < 0.001 compared with untreated rats at the same gestational day

In the basal zone, the protein levels of Mas1 and MME were significantly higher in the DEX group at 21 dg compared to 19 dg (*p* < 0.01; Fig. 4a & b). In addition, the protein levels of Mas1 and MME were significantly higher in the DEX group compared to the control group at 21 dg (*p* < 0.01, *p* < 0.05, respectively; Fig. 4a & b). ACE2 and Ang-(1–7) proteins were not detected in the basal zone.

In the labyrinth zone, the protein levels of ACE2 and Ang-(1–7) were significantly lower in the DEX group compared to the control group at 21 dg (*p* < 0.05; Fig. 4c & d). The protein levels of Mas1 and MME were significantly higher in the DEX group at 21 dg compared to 19 dg (*p* < 0.05 and *p* < 0.01, respectively; Fig. 4e & f). Moreover, the protein levels of Mas1 and MME were significantly higher in the DEX group compared to the control group at 21 dg (*p* < 0.05 and *p* < 0.001, respectively; Fig. 4e & f).

## Discussion

This is the first study to report the effect of maternal glucocorticoid treatment on the expression of ACE2, MME, Ang-(1–7) and Mas1 receptor in the rat placental zones. This study shows that maternal dexamethasone treatment leads to IUGR and reduces the levels of ACE2 and Ang-(1–7) in the rat labyrinth zone in late pregnancy. These findings suggest that a correlation exists between glucocorticoid treatment, ACE2 and Ang-(1–7) levels in the placental labyrinth zone and impaired fetal growth.

Our findings confirm the role of Ang-(1–7) in normal pregnancy as the expression of its receptor Mas1 and the enzymes involved in its production (ACE2 and MME) were significantly elevated in control rats at 21 dg. This result is consistent with previous findings [27, 34, 35] indicating that the system tends to produce more Ang-(1–7) in late pregnancy possibly in an attempt to cause vasodilation and increase blood flow to the rapidly growing fetus late in gestation.

Our results showed that ACE2 and Ang-(1–7) were not expressed in the maternal side of the placenta (basal zone) late in pregnancy despite their abundance in the fetal side (labyrinth zone). This result is consistent with the previous studies in rats [27] [36] and in humans [37]. Valdes and colleagues [37] showed that Ang-(1–7) and ACE2 staining was clear in the cytotrophoblasts, syncytiotrophoblasts and the endothelium of the blood vessels of the primary and secondary villi of the human placenta in late pregnancy. These regions are equivalent to the labyrinth zone of the placenta in rats. This finding indicates that Ang-(1–7) in the placenta has a significant role in blood flow regulation of the highly vascularized labyrinth zone and a less important role in hormone production which is mainly performed by the basal zone. This autocrine regulation of placental blood flow seems to be altered by the reduced production of ACE2 and Ang-(1–7) through prenatal administration of glucocorticoids leading to IUGR.

Dysregulation of the components of the renin angiotensin system was previously reported in pathological pregnancies. In preeclampsia, lower plasma levels of Ang-(1–7) and persistent elevation of plasma Ang II levels were reported possibly causing vasoconstriction resulting in the high blood pressure in preeclamptic subjects [31]. In addition to plasma levels, elevation in local uterine-placental bed RAS activation was evident in preeclamptic women as shown by an elevation in Ang II, renin and ACE mRNA possibly vasoconstricting fetal chorionic villi vessels leading to decreased maternal-fetal oxygen exchange and fetal nutrition [38]. Involvement of Ang-(1–7) in preterm deliveries is suggested as fetal and maternal plasma Ang-(1–7) concentrations were lower in preterm births (less than 37 gestational weeks) compared to full-term births (37 gestational weeks or more) [39]. In pregnant ACE2-knockout mice, plasma Ang-(1–7) was reduced whereas placental and renal Ang II levels were elevated. These changes were associated with decreased gestational body weight gain, reduced fetal weight and length as well as an elevation in maternal blood pressure [40]. Another factor that causes reduced fetal growth and program adulthood diseases is the maternal protein restriction. Gao and colleagues reported reduced levels of ACE2 in the placental labyrinth zone dissected from dams subjected to low protein diet [35]. Collectively, the results of these studies suggest that the ratio between Ang II and Ang-(1–7) is more important than individual levels to determine the degree of vascular tone, blood flow and consequently nutrient supply to the fetus in late pregnancy.

Although glucocorticoid administration prevented the rise in Ang-(1–7) and ACE2 levels in the placental labyrinth zone at late gestation (21dg), to our surprise, the protein expression of MME and Mas1 receptor was significantly elevated in the DEX-treated rats. The increase in MME expression may suggest an elevation in the alternative pathway to compensate for the reduced production of Ang-(1–7) through the major production pathway (ACE2 pathway). Similarly, a normal consequence of the reduced ligand availability is the up-regulation of the receptors to enhance the sensitivity of the receptors to the ligand. Therefore, Mas1 receptors were up-regulated in the glucocorticoid treated rats at late gestation but how significant was this increase in terms of reducing the degree of IUGR is a question that cannot be answered by the experimental settings of the current study.

## Conclusions

This study is the first to find an association between the administration of glucocorticoids and alterations of ACE2 and Ang-(1–7) levels in the labyrinth zone of the placenta in late pregnancy. These results suggest that the drop in ACE2 and/or Ang-(1–7) production may be responsible for a reduced placental blood flow prompting the fetus to compromised nutrient supply and eventually growth restriction.

### Perspectives

This study opens a wide area of research to further explore the molecular pathways involved in the regulation of placental blood flow in glucocorticoid treated mothers. To confirm the role of ACE2/Ang-(1–7)/Mas1 receptor axis in fetal growth and development, Mas1 receptor antagonists and ACE2 inhibitors could be used. This research may also support the postulated therapeutic role of ACE2/Ang-(1–7)/Mas1 receptor axis where recombinant ACE2, Ang-(1–7) analogue (AVE0991) and the Mas1 receptor agonist (CGEN-856S) may be used alongside with glucocorticoids to recover blood flow and prevent IUGR.
