# Expression of Concern to: The role of ACE2, angiotensin-(1–7) and Mas1 receptor axis in glucocorticoid-induced intrauterine growth restriction

**DOI:** 10.1186/s12958-020-00609-9

**Published:** 2020-05-25

**Authors:** Elham Ghadhanfar, Aseel Alsalem, Shaimaa Al-Kandari, Jumana Naser, Fawzi Babiker, Maie Al-Bader

**Affiliations:** 1 Department of Physiology, Faculty of Medicine, Kuwait University, Kuwait City, Kuwait; 2 Faculty of Medicine, Kuwait University, Kuwait City, Kuwait

## Abstract

An amendment to this paper has been published and can be accessed via the original article.

**Expression of Concern to: Reprod Biol Endocrinol 15, 97 (2017)**

**https://doi.org/10.1186/s12958-017-0316-8**

The Editors-in-Chiefare issuing an Expression of Concern for this article [1]. After publication concerns were raised with respect to the specificity and validity of the placental Ang-(1–7) levels assessed by Western blotting and reported in Fig. 4 because Ang-(1–7), which is less than 1 kDa, was detected as a 34 kDa protein. All authors disagree with this Expression of Concern.
